# Comparison of Renal Tubular Damage with Kidney Injury Molecule-1 in Open and Laparoscopic Colorectal Cancer Surgery

**DOI:** 10.3390/medicina61010042

**Published:** 2024-12-30

**Authors:** Abdullah Gürhan Duyan, Celalettin Vatansev, Rahim Kocabaş, Melek Yalçın Koç, Muhammed Ali Akbulut

**Affiliations:** 1 Konya City Hospital, Konya 42020, Turkey; dr.maliakbulut@gmail.com; 2 Faculty of Medicine, Necmettin Erbakan University, Konya 42090, Turkey; cvatansev@erbakan.edu.tr; 3 Faculty of Medicine, Karamanoğlu Mehmetbey University, Karaman 70100, Turkey; drrahim42@gmail.com; 4 Beyhekim Training and Research Hospital, Konya 42060, Turkey; melekyalcin37@gmail.com

**Keywords:** kidney injury molecule-1, laparoscopic colon and rectal surgery, renal tubular damage

## Abstract

*Background and Objectives*: Colorectal cancer is the third most common type of cancer in men and women. With advancements in technology, minimally invasive treatment options have become increasingly prominent in colorectal cancer surgery. This study aimed to compare the increased intra-abdominal pressure in laparoscopic colon and rectal surgery with open procedures using kidney injury molecule-1 (KIM-1) secreted from renal tubules. *Materials and Methods*: We enrolled 46 patients diagnosed with colon cancer who underwent laparoscopic and open surgical procedures at our clinic. The patients were prospectively randomized into five groups: 10 laparoscopic right hemicolectomies (Group 1), 8 open right hemicolectomies (Group 2), 8 laparoscopic anterior resections (LARs) (Group 3), 11 open anterior resections (Group 4), and 9 laparoscopic low anterior resections (Group 5). Urine samples were collected from the patients preoperatively, postoperatively at the 4th hour, and postoperatively on the 14th day, and the urine KIM-1 levels and urine creatinine (Cr) values were measured. The urine KIM-1/Cr ratios were subsequently calculated. *Results*: The urinary KIM-1/Cr levels increased at the 4th postoperative hour after the open and laparoscopic procedures. On postoperative day 14, the urinary KIM-1/Cr levels were lower than those in the preoperative period in all groups, except the LAR group. *Conclusions*: Our study shown that the average pressure in laparoscopic colon and rectal surgery did not have a long-term impact on kidney injury in comparison to open colon and rectal surgery.

## 1. Introduction

Colorectal cancer (CRC) is a malignancy of the large intestine. CRC usually develops as a result of the progressive malignant transformation of epithelial tissues [1]. Tumors that develop in this manner are called adenocarcinomas and account for most colorectal carcinomas [2]. As of 2020, CRC is the third most common cancer worldwide and the second most common cause of cancer-related deaths [3]. Ongoing global demographic and epidemiological changes continue to increase the burden of cancers on healthcare systems, and it is estimated that the number of new CRC cases will increase to 2.2 million and that annual deaths due to CRC will increase to 1.1 million by 2030 [4].

New surgical techniques in colorectal surgeries allow for the removal of organs affected by the primary tumor, including the regional lymph nodes, guided by embryological plans. There are studies comparing laparoscopic and robotic techniques with similar oncologic and functional outcomes [5]. Conversely, numerous studies indicate that robotic surgery surpasses laparoscopic surgery [6,7,8]. But, in robotic and laparoscopic procedures, intra-abdominal insufflation of carbon dioxide is needed to provide adequate visualization of the surgical field. The kidneys may be especially sensitive to intra-abdominal pressure [9], and both animal and human studies suggest that even short durations of slightly increased intra-abdominal pressure can impair renal function [10,11]. Although surgery is the mainstay of treatment, adjuvant or neoadjuvant systemic chemotherapy, radiotherapy, and molecular targeted agents are also used, depending on the patient’s clinical characteristics [12]. Research indicates that total neoadjuvant therapy for rectal cancer can yield a significant rate of pathologic full responses in patients [13,14].

With the development of technology in colorectal cancer surgery, minimally invasive treatment options have come to the forefront. This study aimed to compare the intra-abdominal pressure created in laparoscopic colorectal surgery with that in open procedures by studying the kidney injury molecule-1 (KIM-1) secreted from the renal tubules in the urine, which is the most sensitive response to renal damage, and urinary creatinine (CR). This study is the first research in the literature to assess renal damage utilizing the KIM-1 molecule in both laparoscopic and open colorectal surgeries.

### 1.1. Role of Kidney Injury Molecule-1 (KIM-1)

KIM-1 is a type 1 cell membrane glycoprotein containing N and O glycosylation sites and exhibiting extracellular immunoglobulin and mucin-like effects [15]. Renal tubule epithelial cells undergo apoptosis after injury for various reasons. Apoptotic and necrotic cells must be cleared to reduce inflammation and ensure tissue repair. KIM-1 is a phagocytic phosphatidylserine receptor found in the renal epithelial cells. It specifically recognizes phosphatidylserine epitopes on the surface of apoptotic tubular epithelial cells [16]. The level of KİM-1 in urine increases when renal damage increases in a mouse model [17].

### 1.2. Relationship Between Acute Kidney Injury and KIM-1

Acute kidney injury (AKI) is defined as a ≥50% increase in the baseline plasma Cr concentration within 7 days and/or a 0.3 mg/dL increase in the serum Cr level within 2 days. KIM-1 messenger RNA is highly expressed during proximal tubule epithelial cell renewal. It is upregulated in the S3 segment of the proximal tubule after ischemia, and the S3 segment is highly sensitive to ischemia [18]. KIM-1 is expressed on the apical side of proximal tubule epithelial cells but not in the glomeruli with acute tubular necrosis. After ischemic kidney injury, KIM-1 is rapidly expressed in the urine 12 h before the regeneration of the epithelium. According to an experiment in mice, urinary KIM-1 levels increased 16-, 48-, and 60-fold after 10, 20, and 30 min of ischemia in the kidney, respectively, compared with the control group. Sabbisetti et al. found that the plasma concentration of KIM-1 was positively correlated with corrected urinary KIM-1 (r = 0.43, *p* < 0.001) and uncorrected urinary KIM-1 (r = 0.24, *p* = 0.02) levels [17]. KIM-1 levels increase not only in AKI but also in chronic processes, such as polycystic kidney disease, renal cell carcinoma, and chronic renal failure [19]. The European Food and Drug Administration has approved KIM-1 as a highly sensitive and specific urinary biomarker for monitoring drug-induced kidney injury in both clinical and preclinical studies [20].

## 2. Materials and Methods

This study was approved by the Ethics Committee of Necmettin Erbakan University Faculty of Medicine (number 2021/3475). Verbal and written informed consent was obtained from all the study participants.

Between November 2021 and May 2022, urine samples were collected from 46 patients with CRC and prospectively evaluated at the Department of General Surgery, Faculty of Medicine, Necmettin Erbakan University.

Forty-six patients who were admitted to the general surgery clinic of our hospital between November 2021 and May 2022, were diagnosed with colorectal cancer, and underwent laparoscopic and open procedures were included in our study. The patients were prospectively randomized into five groups: 10 laparoscopic right hemicolectomies (LRHs) (Group 1), 8 open right hemicolectomies (RHs) (Group 2), 8 laparoscopic anterior resections (LARs) (Group 3), 11 open anterior resections (Group 4), and 9 laparoscopic low anterior resections (LLARs) (Group 5). Urine samples were collected from the patients preoperatively, at the 4th hour postoperatively, and 14 days postoperatively, and the urine KIM-1 levels and creatinine levels were measured. The urine KIM-1/Cr ratios were subsequently calculated.

In LRHs and RHs, the operating table was positioned neutrally. In the other groups, the position was Trendelenburg and inclined at 25 degrees. The 46 patients included in this study were selected from among those who underwent surgery for colorectal tumors at our hospital and had no distant metastases. The ratios of males to females were similar. The patients did not receive any neoadjuvant treatment. Patients who underwent emergency surgery or had any known comorbidities were excluded. The patients were not administered preoperative, intraoperative, or postoperative nephrotoxic drugs. The patients had no known chronic diseases. Voluntary individuals without cognitive impairments were included in this study. The study adhered to the guidelines of the Declaration of Helsinki. All participants were provided with a “Patient Information Form” and “Patient Consent Form”, which were read to them, and their signed consent was obtained.

### 2.1. Measurement of KIM-1/Creatinine Levels and Storage of Samples

Preoperative, postoperative 4th hour, and postoperative 14th day urine samples were collected from the patients and centrifuged at 3000 rpm for 10 min. The serum samples obtained were divided into Eppendorf tubes and stored at −80 °C until analysis. On the day of the study, the samples were thawed, and the urine KIM-1 levels were measured using specific enzyme-linked immunosorbent assay (ELISA) test kits (Elabscience Biotechnology Co. Ltd, Houston, TX, USA) in accordance with the manufacturer’s instructions. The urinary KIM-1 concentration in each sample was calculated using a calibration graph prepared from the standards. The KIM-1 concentration was measured in pg/mL. The KIM-1/Cr ratios were then calculated and compared among the groups.

### 2.2. Data Analysis

The data obtained as a result of the study were analyzed using the Statistical Package for the Social Sciences version 18.0 program (SPSS, Inc., Chicago, IL, USA).

In descriptive analyses, the frequency data are presented as numbers (n) and percentages (%), and the numerical data are presented as means ± standard deviations.

The compatibility of the numerical data with a normal distribution was analyzed using the Kolmogorov–Smirnov test. The distribution of normally distributed numerical data in the two independent groups was evaluated using the independent-samples *t*-test, and the distribution of non-normally distributed numerical data was evaluated using the Mann–Whitney U test. In the two dependent groups, numerical data conforming to the normal distribution were evaluated using the paired-samples *t*-test, and numerical data not conforming to the normal distribution were evaluated using the Wilcoxon signed-rank test. The Kruskal–Wallis test was used to evaluate numerical data that were non-normally distributed in more than two groups. Post hoc analysis was performed using the Mann–Whitney U test with Bonferroni correction for variables found to be significant in the Kruskal–Wallis test.

The relationship between non-normally distributed numerical data was evaluated using Spearman correlation analysis. A correlation relationship of r = 0.05–0.30 was considered low or insignificant, r = 0.30–0.40 was low–moderate, r = 0.40–0.60 was moderate, r = 0.60–0.70 was good, r = 0.70–0.75 was very good, and r = 0.75–1.00 was excellent.

The statistical significance level was set at *p* < 0.05 for all tests.

## 3. Results

This study included 46 patients who underwent colorectal surgery at Necmettin Erbakan University, Medical Faculty Hospital. Of the patients who underwent surgery, 50.0% (n = 23) were male, and 50.0% (n = 23) were female. The mean age was 61.65 ± 11.94 years, and the mean body mass index (BMI) was 26.26 ± 5.34 kg/m^2^. The surgical types of the patients are listed in Table 1.

Laparoscopic surgery was performed in 58.7% (n = 27) of the patients included in this study. The intra-abdominal pressure was 12 mmHg in each patient who underwent laparoscopic surgery. The age, preoperative KIM-1/Cr levels, postoperative 4th hour and postoperative 14th day KIM-1/Cr levels, operation times, and sex ratios were similar between the open and laparoscopic surgery groups (*p* > 0.05) (Table 2).

In patients who underwent laparoscopic surgery, there was a moderate positive correlation between the patient age and preoperative KIM-1/Cr and KIM-1/Cr levels measured postoperatively at the 4th h (r = 0.403 and r = 0.403, with *p* = 0.037 and *p* = 0.037, respectively). In patients who underwent open surgery, there was a negative moderately statistically significant correlation between the BMI and KIM-1/Cr level measured on postoperative day 14 (r = −0.484, *p* = 0.036) (Table 3).

Table 4 shows a comparison of the ages, laboratory values, and operation times of patients who underwent laparoscopic surgery according to the type of operation.

The KIM-1/Cr levels measured on postoperative day 14 in patients who underwent LAR (Table 4) were statistically significantly higher than those in the other laparoscopic operation groups (*p* = 0.022). The preoperative KIM-1/Cr levels were significantly lower in patients who underwent LLAR than in those who underwent RH, AR, LRH, and LAR (*p* = 0.022). At postoperative day 14, the KIM-1/Cr levels were significantly higher in patients who underwent LAR than in those who underwent RH, AR, LRH, and LLAR (*p* = 0.038) (Table 5).

## 4. Discussion

Postoperative AKI is associated with significant morbidity, increased mortality, prolonged hospital and ICU stays, and high healthcare costs [21]. It is, thus, of considerable interest to determine whether peritoneal insufflation combined with Trendelenburg positioning impairs renal function [22]. Although there were convincing reasons to expect an association between the surgical approach and renal injury, none was observed in our analysis. A laparoscopic surgical approach was not associated with a reduction in postoperative urinary KIM-1/Cr. Consequently, the choice of surgical approach (open vs. laparoscopic) should not be influenced by the attempt to prevent renal injury. Well-known benefits of the laparoscopic surgical approach, including reduced blood loss, decreased postoperative pain, and shorter in-hospital recovery, should be considered instead. Given the other advantages of laparoscopic surgery, the approach should not be avoided for concerns about renal injury.

The study conducted by Khalil et al. involved the analysis of blood samples to assess the hemoglobin A1c (HbA1c) (%), urea nitrogen, uric acid, creatinine, and KIM-1 levels before and six months following laparoscopic sleeve gastrectomy in 44 morbidly obese individuals. Six-month post-surgery data showed significant reductions in the body mass index, HbA1c, microalbuminuria, and serum KIM-1 and a significant increase in eGFR (all, *p* < 0.001) [23]. In our study, we compared the open and closed variants of identical procedures and sought to evaluate the damage inflicted directly by laparoscopic surgery on renal tubule epithelial cells through KIM-1/Cr in preoperative and early postoperative urine samples.

Sim, J H et al. retrospectively compared the incidence of postoperative AKI between laparoscopic and open surgery in 3637 patients with colorectal cancer. Pre- and postoperative laboratory values included the estimated glomerular filtration rate (eGFR), creatinine, and serum albumin. The results of the study demonstrated no significant difference in the overall postoperative AKI incidence between laparoscopic and open surgeries (8.8% vs. 9.1%) in CRC [24]. Our study has shown that kidney damage resulting from elevated intra-abdominal pressure after laparoscopic colorectal cancer surgery is similar to that observed in open colorectal cancer surgery.

Increased intra-abdominal pressure is associated with decreased renal blood flow, renal hypoxia, and acute kidney injury. H. Essber, B. Cohen, and A.S. Artis et al. compared that when combined with Trendelenburg positioning, renal function may further deteriorate. A total of 1846 laparoscopic cases with Trendelenburg positioning were matched to 1846 open cases. No association was found between the laparoscopic approach and postoperative eGFR. Patients who had laparoscopic surgeries were an estimated 0.70 (95% CI 0.55, 0.90, pHolm-adj = 0.006) times as likely to have AKI as open surgical patients. Despite compelling potential mechanisms, laparoscopic approach with the Trendelenburg position in adult colorectal surgeries did not worsen postoperative eGFR and actually reduced the incidence of postoperative acute kidney injury [25]. In our study, right colon surgeries were performed in the neutral position and left colon and rectal surgeries were performed in the Trendelenburg position. In the early postoperative period, the urinary KIM-1/Cr values increased in all patients. On the 14th postoperative day, the urinary KIM-1/Cr values decreased to preoperative levels in all patients. Our study also showed that both increased intra-abdominal pressure and the Trendelenburg position did not cause permanent renal damage.

In addition, the role of KIM-1 in determining kidney injury has been investigated in studies investigating many different kidney injuries.

In a study conducted by Peng et al., urinary KIM-1 levels increase with age in healthy individuals, and a model created using KIM-1 can predict the renal age in healthy individuals [26]. In our study, there was a moderate positive correlation between the patient age and preoperative KIM-1/Cr and KIM-1/Cr levels measured at the 4th hour postoperatively in patients who underwent laparoscopic surgery (r = 0.403 and r = 0.403, with *p* = 0.037 and *p* = 0.037, respectively).

Xu et al. analyzed the plasmas of 418 patients. Higher KIM-1 levels after nephrectomy for renal cell carcinoma were associated with worse overall survival after multivariable adjustment. The plasma KIM-1 level after nephrectomy in patients with renal cell carcinoma was examined, and it was shown to be a statistically significant biomarker for residual disease [27].

In research conducted by Bonventre et al., KIM-1 proved to be an outstanding marker of renal damage in rats, outperforming BUN and serum Cr as markers of histopathological changes in the proximal tubule in response to several pathophysiological conditions or toxic substances. Tissue expression and urinary excretion of the ectodomain of KIM-1 are sensitive and specific damage markers and outcome predictors [28]. In our study, to ensure standardization, the same anesthetic agents were administered to the patients in correlation with the operation time, and the same or similar medical treatments were administered during the postoperative period.

Duan et al. investigated whether urinary KIM-1, interleukin-18 (IL-18), and Cys-C are early predictive biomarkers of gadolinium-based contrast-induced nephropathy (Gd-CIN) in elderly patients undergoing gadolinium-enhanced magnetic resonance imaging (MRI). Urinary KIM-1, IL-18, and Cys-C are early predictive biomarkers of Gd-CIN in elderly patients and perform better than serum Cr for the early diagnosis of Gd-CIN [29]. In this study, the acute toxicity of gadolinium contrast medium in the kidney was studied using KIM-1 and was found to be significant.

Kwon et al. demonstrated that tissue KIM-1 expression was associated with renal outcomes in immunoglobulin A (IgA) nephropathy. The results of multivariate regression analysis were consistent with the association between tissue KIM-1 levels and prognosis. The urinary KIM-1/Cr concentration was significantly higher in patients with IgA nephropathy than in normal controls [30].

In a study by Hussein et al., KIM-1 was studied in lead nephropathy, and renal side effects were found to be associated with lead smoke exposure in 35 patients [31]. In this study, chronic toxicity damage was studied using KIM-1, and urinary KIM-1/Cr levels during surgery were assessed.

Polidori et al. compared kidney damage in 40 obese prepubertal children with 29 healthy age- and sex-matched peers. The NGAL and KIM-1 levels were higher in obese children than in controls. KIM-1 demonstrates that obese participants exhibit a certain degree of renal damage before the loss of renal function [32]. In our study, no significant differences were found between the BMI and urinary KIM-1/Cr values preoperatively, at the 4th h postoperatively, and on the 14th day postoperatively in patients undergoing laparoscopic surgery. However, in patients who underwent open surgery, a moderately significant negative correlation was found between the VKI and KIM-1/Cr levels measured on postoperative day 14 (r = −0.484, *p* = 0.036).

As shown in these studies, KIM-1 can be used as a biomarker for detecting renal tubule damage. It can be used in the diagnosis of acute and chronic renal pathologies and the detection of recurrent cases of renal carcinomas. Several studies have reported the use of KIM-1 in different pathologies, but no studies have compared laparoscopic and open surgeries. To the best of our knowledge, this is the first such study in this field. In this study, we investigated the effects of trauma caused by intra-abdominal pressure on renal proximal tubule cells during laparoscopic surgery. The KIM-1/Cr value measured in the urine at the 4th hour postoperatively was significantly increased in all groups. Although this increase was thought to be due to an increase in intra-abdominal pressure, the unexpected increase in the number of open surgeries led us away from this idea. On postoperative day 14, the urinary KIM-1/Cr values significantly decreased in all patients, except those in the LAR group. No reason was found to explain the high urinary KIM-1/Cr ratio in the LAR group on postoperative day 14. In this context, it was observed that trauma due to increased intra-abdominal pressure caused by laparoscopic surgery was not statistically significant compared with open surgery and did not cause permanent kidney damage.

Our study has some limitations. Research on KIM-1 expression in patients after neoadjuvant therapy may elucidate kidney damage. The study design was a single-center prospective study, and generalization of the findings requires confirmation in larger studies. Additionally, the sample size was relatively small. Therefore, further studies with larger sample sizes are required.

## 5. Conclusions

KIM-1/Cr measured in the urine is a sensitive biomarker for kidney injury. Increased intra-abdominal pressure during laparoscopic surgery may cause functional changes and damage various abdominal organs. Our study showed that the mean pressure in laparoscopic colon and rectal surgery had no long-term effect on renal injury compared with open colon and rectal surgery. However, we believe that different results may be obtained in clinical studies with appropriate patient numbers, higher intra-abdominal pressures, and longer operative times.

## Figures and Tables

**Table 1:** Distribution of patients’ operation types.

Operation Types	n (%)
Right hemicolectomy	8 (17.4)
Anterior resection	11 (23.9)
Laparoscopic right hemicolectomy	10 (21.7)
Laparoscopic anterior resection	8 (17.4)
Laparoscopic low anterior resection	9 (19.6)

**Table 2:** Comparison of laboratory values and operation times according to the type of surgery.

	Open Surgery (n = 19) Average ± Standard Deviation (SD)	Laparoscopic Surgery (n = 27) Average ± SD	*p*
Age	60.10 ± 14.63	62.74 ± 9.77	0.499
Kidney injury molecule-1 (KIM-1)/creatinine (Cr) preop	0.17 ± 0.19	0.14 ± 0.10	0.680
KIM-1/Cr postop 4 h	0.33 ± 0.41	0.23 ± 0.14	0.260
KIM-1/Cr postop 14 days	0.10 ± 0.06	0.10 ± 0.08	0.973
Operation duration (min)	134.47 ± 32.86	112.66 ± 46.65	0.231
Sex, n (%)			
Female	11 (57.9)	12 (44.4)	0.369
Male	8 (42.1)	15 (55.6)	

**Table 3:** Relationship between kidney injury molecule-1 (KIM-1) values and patient ages and body mass index (BMI) levels in patients undergoing laparoscopic and open surgery.

		Variables	r	*p*
Laparoscopic surgery	Age	Preop KIM-1/Cr	**0.403**	**0.037**
		Postop KIM-1/Cr 4 h	**0.403**	**0.037**
		Postop KIM-1/Cr 14 days	0.090	0.655
	BMI (kg/m^2^)	Preop KIM-1/Cr	−0.014	0.943
		Postop KIM-1/Cr 4 h	−0.268	0.177
		Postop KIM-1/Cr 14 days	−0.274	0.166
Open surgery	Age	Preop KIM-1/Cr	−0.036	0.884
		Postop KIM-1/Cr 4 h	0.159	0.516
		Postop KIM-1/Cr 14 days	−0.029	0.906
	BMI (kg/m^2^)	Preop KIM-1/Cr	0.286	0.235
		Postop KIM-1/Cr 4 h	0.226	0.353
		Postop KIM-1/Cr 14 days	**−0.484**	**0.036**

KIM-1: Kidney Injury Molecule-1, BMI: Body Mass Index, Cr: Creatinine, Preop: Preoperative, Postop: Postoperative, h: hour.

**Table 4:** Comparison of laboratory values and operation times in patients undergoing laparoscopic surgery.

	Laparoscopic Right Hemicolectomy (n = 10) Average ± Standard Deviation (SD)	Laparoscopic Anterior Resection (n = 8) Average ± SD	Laparoscopic Low Anterior Resection (n = 9) Average ± SD	*p*
Age	60.50 ± 12.54	65.75 ± 9.96	62.55 ± 5.59	0.544
KIM-1/Cr preop	0.18 ± 0.13	0.18 ± 0.10	0.07 ± 0.02	0.055
KIM-1 Cr postop 4 h	0.25 ± 0.14	0.26 ± 0.14	0.18 ± 0.14	0.459
KIM-1/Cr postop 14 days	0.08 ± 0.04	0.17 ± 0.12 *	0.06 ± 0.04	**0.022**
Operation duration (min)	108.50 ± 23.10	116.25 ± 46.27	140.00 ± 47.16	0.224
Sex, n (%)				
Female	3 (30.0)	6 (75.0)	3 (33.3)	
Male	7 (70.0)	2 (25.0)	6 (66.7)	

* The group in which the difference originated.

**Table 5:** Comparison of body mass indexes (BMIs), kidney injury molecule-1 (KIM-1)/creatinine (Cr) values, and operation times in patient groups.

	Right Hemicolectom y (n = 8) Average ± Standard Deviation (SD)	Anterior Resection (n = 11) Average ± SD	Laparoscopic Right Hemicolectomy (n = 10) Average ± SD	Laparoscopic Anterior Resection (n = 8) Average ± SD	Laparoscopic Low Anterior Resection (n = 9) Average ± SD	*p*
BMI (kg/m^2^)	24.88 ± 7.57	24.50 ± 5.09	26.49 ± 4.50	28.56 ± 7.57	27.33 ± 5.96	0.480
KIM-1/Cr preop	0.27 ± 0.25	0.09 ± 0.07	0.18 ± 0.13	0.18 ± 0.10	0.07 ± 0.02 *	**0.022**
KIM-1/Cr postop 4 h	0.37 ± 0.22	0.29 ± 0.52	0.25 ± 0.14	0.26 ± 0.14	0.18 ± 0.14	0.743
KIM-1/Cr postop 14 days	0.11 ± 0.08	0.09 ± 0.04	0.08 ± 0.04	0.17 ± 0.12 *	0.06 ± 0.04	**0.038**
Operation duration (min)	115.62 ± 30.64	148.18 ± 28.21	108.50 ± 23.10	116.25 ± 46.27	140.00 ± 47.16	0.072

* The group in which the difference originated.

## Data Availability

The data presented in this study are available on request from the corresponding author due to ethical reasons.
